# Antenatal Multiple Micronutrient Supplementation Compared to Iron–Folic Acid Affects Micronutrient Status but Does Not Eliminate Deficiencies in a Randomized Controlled Trial Among Pregnant Women of Rural Bangladesh

**DOI:** 10.1093/jn/nxz046

**Published:** 2019-04-22

**Authors:** Kerry J Schulze, Sucheta Mehra, Saijuddin Shaikh, Hasmot Ali, Abu Ahmed Shamim, Lee S-F Wu, Maithilee Mitra, Margia A Arguello, Brittany Kmush, Pongtorn Sungpuag, Emorn Udomkesmelee, Rebecca Merrill, Rolf D W Klemm, Barkat Ullah, Alain B Labrique, Keith P West, Parul Christian

**Affiliations:** 1 Center for Human Nutrition, Department of International Health, Johns Hopkins Bloomberg School of Public Health, Baltimore, MD; 2 The JiVitA Project of Johns Hopkins University, Bangladesh, Gaibandha, Bangladesh; 3 Institute of Nutrition, Mahidol University, Bangkok, Thailand

**Keywords:** micronutrients, vitamins, minerals, pregnancy, antenatal, supplementation, trial, Bangladesh, South Asia

## Abstract

**Background:**

Antenatal multiple micronutrient (MM) supplementation improves birth outcomes relative to iron–folic acid (IFA) in developing countries, but limited data exist on its impact on pregnancy micronutrient status.

**Objective:**

We assessed the efficacy of a daily MM (15 nutrients) compared with IFA supplement, each providing approximately 1 RDA of nutrients and given beginning at pregnancy ascertainment, on late pregnancy micronutrient status of women in rural Bangladesh. Secondarily, we explored other contributors to pregnancy micronutrient status.

**Methods:**

Within a double-masked trial (JiVitA-3) among 44,500 pregnant women, micronutrient status indicators were assessed in *n* = 1526 women, allocated by cluster to receive daily MM (*n* = 749) or IFA (*n* = 777), at 10 wk (baseline: before supplementation) and 32 wk (during supplementation) gestation. Efficacy of MM supplementation on micronutrient status indicators at 32 wk was assessed, controlling for baseline status and other covariates (e.g., inflammation and season), in regression models.

**Results:**

Baseline status was comparable by intervention. Prevalence of deficiency among all participants was as follows: anemia, 20.6%; iron by ferritin, 4.0%; iron by transferrin receptor, 4.7%; folate, 2.5%; vitamin B-12, 35.4%; vitamin A, 6.7%; vitamin E, 57.7%; vitamin D, 64.0%; zinc, 13.4%; and iodine, 2.6%. At 32 wk gestation, vitamin B-12, A, and D and zinc status indicators were 3.7–13.7% higher, and ferritin, γ-tocopherol, and thyroglobulin indicators were 8.7–16.6% lower, for the MM group compared with the IFA group, with a 15–38% lower prevalence of deficiencies of vitamins B-12, A, and D and zinc (all *P* < 0.05). However, indicators typically suggested worsening status during pregnancy, even with supplementation, and baseline status or other covariates were more strongly associated with late pregnancy indicators than was MM supplementation.

**Conclusions:**

Rural Bangladeshi women commonly entered pregnancy deficient in micronutrients other than iron and folic acid. Supplementation with MM improved micronutrient status, although deficiencies persisted. Preconception supplementation or higher nutrient doses may be warranted to support nutritional demands of pregnancy in undernourished populations. This trial was registered at clinicaltrials.gov as NCT00860470.

## Introduction

Micronutrients support vital aspects of maternal and fetal health during pregnancy (1). We recently reported results from JiVitA-3, a cluster-randomized trial of daily antenatal multiple micronutrient (MM) compared with iron–folic acid (IFA) supplementation in >44,000 pregnant women in rural Bangladesh (2). There was an 11% decline in stillbirths, a 15% decline in preterm births, a 12% decline in low birth weight, and a nonsignificant decline in small-for-gestational-age births with the MM intervention compared with IFA (2). An average increase in gestational age of 0.3 wk in the MM recipients was associated with higher birth weight (54 g) and corresponding improvements in infant length and head, arm, and chest circumferences (2). A recent meta-analysis comparing antenatal MM to IFA in developing countries showed that MM formulations significantly improved birth outcomes, prompting the authors to encourage MM supplement use in settings in which micronutrient deficiencies exist (3).

Multiple micronutrient supplements typically contain ∼1 RDA of vitamins and minerals and are intended to fill nutritional gaps assumed to be common during pregnancy (4). Deficiencies are often inferred from dietary data (5–7) because limited biochemical data exist on the nutritional burdens of pregnant women beyond anemia and, less frequently, iron, vitamin A, folate, zinc, or iodine status (8). We previously showed in Nepal that women entered pregnancy deficient in numerous micronutrients (9), with most deficiencies responding to MM supplementation (10, 11). But the impact of MM supplements could vary by context, and an ∼1 RDA boost in micronutrients may be insufficient to meet demands of pregnancy where nutritional, infectious, and environmental stresses prevail. More comprehensive data on micronutrient status could help fill gaps in our knowledge of need for and benefits of supplementation and risks of deficiencies to mother and fetus under varied population conditions. Given the essential roles of micronutrients in supporting both maternal and fetal functions over a reproductive event (1), more research on maternal status during pregnancy and the impact of MM supplementation on the status of populations at risk of deficiencies is a critical need.

Assessing micronutrient status during pregnancy is challenging, however. Among women in the JiVitA-3 trial in Bangladesh, we determined that plasma volume expanded <15% as women gained ∼6 kg from 10 to 32 wk gestation (12), compared to estimates by others of ∼40% plasma volume expansion (13). Such expansion may reduce concentrations of micronutrient status indicators through dilution, compounded by nutrient utilization or transfer to the fetus. Consequently, declines over pregnancy in hemoglobin (Hb), ferritin (14), folate and vitamin B-12 (15), and retinol (16, 17) have been shown to occur in the absence of supplementation. Functional indicators such as transferrin receptor (TfR) (18) or thyroglobulin (Tg) (19), reflecting demand for iron and iodine, respectively, may remain stable or increase depending on the availability of those nutrients. α-Tocopherol (vitamin E) increases during pregnancy with changes in lipid metabolism (20), whereas vitamin D status may be more affected by factors such as season (21). Pregnancy-specific cutoffs to account for these scenarios are not conventional despite the relevance of pregnancy micronutrient status assessment to maternal and fetal health. Furthermore, more data are required across populations to establish the impact of MM supplementation in relation to pregnancy-associated changes in indicator values.

Here, we assess the efficacy of a daily, antenatal MM (15 nutrients) compared with IFA supplement providing ∼1 RDA of nutrients on late pregnancy micronutrient indicators in pregnant rural Bangladeshi women from the JiVitA-3 trial (2). We describe micronutrient status at entry into pregnancy (∼10 wk gestation; baseline) and in late pregnancy (32 wk gestation) after 5–6 mo of supplementation, hypothesizing a benefit in late pregnancy of the MM intervention for micronutrients other than iron and folate. In addition, we explore pregnancy-associated changes in micronutrient status indicators and the contribution of the MM intervention relative to baseline status and other covariates (e.g., the presence of inflammation and season and gestational age of blood draw) to late pregnancy micronutrient status. This work adds to an understanding of micronutrient deficiencies during pregnancy in a nutritionally compromised population and the ability of antenatal supplementation to address them.

## Methods

### Study design and population

A description of the population research site in Gaibandha District in northwest Bangladesh, design, randomization scheme, field methods, and outcomes of the double-masked, cluster-randomized, controlled trial into which this study was embedded has been provided by West et al. (2). Briefly, 596 mapped community clusters (sectors) of 250–400 geographic information system-addressed households were randomly assigned for women, when pregnant, to receive daily an IFA (standard of care) or MM (15 nutrients) supplement. Married women aged 12–45 y were monitored by local field staff during 5-weekly home visits for menstrual history. Amenorrheic women with pregnancies confirmed by urine test were recruited; consented; and provided with assigned, coded supplements weekly and followed for compliance and pregnancy outcomes. Interviews were conducted in participants’ homes in early pregnancy (baseline), at 32 wk gestation, at birth, and at intervals postpartum to assess factors including household and maternal socioeconomics and demographics, food intake frequencies, and 7-d morbidity history. Socioeconomic variables were combined into a living standards index (LSI) for analysis (22). Recruitment into the study began in January 2008, and the last follow-up of infants was conducted in August 2012, with >127,000 women under pregnancy surveillance, 44,567 confirmed pregnancies, and 28,516 births assessed (2). More intensive data collection from which biospecimens for this analysis were derived took place in a “substudy” (described later), with baseline assessments conducted from June 2008 to May 2010 and participants followed until 3 mo postpartum.

The IFA supplement contained 27 mg iron and 600 µg folic acid, and the MM supplement was similar in appearance and contained identical amounts of iron and folic acid plus vitamins A (770 µg retinol activity equivalents), D (5 µg, 200 IU), E (15 mg), B-1 (thiamin, 1.4 mg), B-2 (riboflavin, 1.4 mg), B-3 (niacin, 18 mg), B-6 (1.9 mg), B-12 (2.6 µg), and C (85 mg) and zinc (12 mg), copper (1 mg), selenium (60 µg), and iodine (220 µg), as recommended by UNICEF (4). Adherence was calculated as the percentage of supplements provided that were reported as consumed from enrollment through 12 wk postpartum, and >50% of women in the trial consumed a median of 95% of distributed supplements (2). Supplements were provided as a premix by DSM, manufactured into tablets by Beximco Pharmaceuticals, stored in opaque bottles in a temperature- and humidity-controlled environment until use, and tested periodically by an independent laboratory to ensure nutrient content (Medallion Laboratories).

Within the study area, 64 sectors (∼10% of the study area) in a centrally located, contiguous area, readily accessible yet generally representative of the larger site, were identified for a substudy that included more extensive data collection at baseline (prior to supplement distribution), 32 wk gestation, and 3 mo postpartum. Blood (7 mL) was collected by venipuncture into sodium heparin trace element-free evacuated tubes by trained phlebotomists during home visits. Hemoglobin (Hb) was assessed immediately with an Hb301 hemoglobinometer (Hemocue). Women with severe anemia (Hb <70 g/L; 1 case at baseline and 3 cases at 32 wk gestation) were offered iron drops and retained in the study. Whole-blood samples were centrifuged in a field laboratory to separate plasma, which was stored in 4 aliquots in liquid nitrogen until shipped in vapor shippers to either the Institute of Nutrition at Mahidol University in Thailand or Johns Hopkins University in Baltimore, Maryland, for analysis.

All procedures were approved by institutional review boards at Johns Hopkins University and the Bangladesh Medical Research Council in Dhaka, Bangladesh.

### Micronutrient status determination and interpretation

We used baseline and 32-wk gestational age samples collected from *n* = 1526 women who completed serial substudy visits (including a delivery visit for cord blood and/or postpartum sample that are not presented here). Data in a small subset of these women (*n* = 80) were previously published but not analyzed by intervention (23).

Samples shipped to the Institute of Nutrition at Mahidol University were assessed by HPLC for retinol, α- and γ-tocopherol, and carotenoids including β-carotene using an adaptation of common methods (24) validated using SRM 968d (National Institute of Standards and Technology). Total cholesterol was assessed using spectrophotometric commercial assay kits (Infinity; ThermoScientific).

At Johns Hopkins University, assays for ferritin, folate, total cobalamin (vitamin B-12), and Tg (for iodine status) were conducted with automated chemiluminescent immunoassays (Immulite 1000 or 2000; Siemens Diagnostics). 25-Hydroxyvitamin D [25(OH)D] (IDS) and transferrin receptor (TfR) (Ramco Labs) were assessed by immunoassay, and α_1_-acid glycoprotein (AGP) was assessed for inflammation using a radial immunodiffusion kit (Kent Laboratories). Plasma zinc was assessed using graphite furnace atomic absorption spectroscopy (AAnalyst 800; PerkinElmer), validated against SRM 1598 (National Institute of Standards and Technology) and run with standards produced from lyophilized human serum (Seronorm; SERO). Typical CVs have been reported (25). Technical staff were blind to intervention status.

Cutoffs to identify micronutrient-deficient women were as follows: Hb <110 g/L (for anemia), ferritin <15 µg/L, TfR >8.3 mg/L (26) for iron status; plasma folate <6.8 nmol/L and cobalamin (B-12) <150 pmol/L (27); retinol <0.70 and <1.05 µmol/L for deficient or marginal vitamin A status, respectively (28), and β-carotene <0.09 µmol/L (24); α-tocopherol <12.0 µmol/L and α-tocopherol:cholesterol <2.2 µmol/mmol (20, 29) for vitamin E; 25(OH)D <50 nmol/L for vitamin D (30); plasma zinc <9.0 µmol/L (10); and Tg >40 µg/L for iodine status (31). Inflammation was assessed as AGP >1.0 g/L (25). Number of deficiencies experienced by participants was calculated using 9 of the indicators: Hb, ferritin, folate, cobalamin, retinol <0.70 µmol/L, α-tocopherol, 25(OH)D, plasma zinc, and Tg. Outlying data points (retinol >2.5 µmol/L, *n* = 4 at baseline; β-carotene >0.9 µmol/L, *n* = 2 at baseline, *n* = 7 at 32 wk; α-tocopherol >40 µmol/L, *n* = 2 at baseline; α-tocopherol:cholesterol >20 at baseline, *n* = 1, or >100 at 32 wk, *n* = 1; cobalamin >750 pmol/L, *n* = 7 at baseline; zinc >30 µmol/L, *n* = 24 at baseline, *n* = 17 at 32 wk) were considered implausible and dropped. Other cases in which laboratory data were incomplete were due to inadequate plasma volumes or, for TfR, a decision to discontinue the assay given expense and little evidence of iron deficiency.

### Data analysis

Baseline characteristics of participants were tested for comparability between the IFA and MM groups by chi-square analysis for categorical variables or by *t* test for continuous variables. In addition, they were tested between substudy participants and the larger trial population to determine the representativeness of the substudy participants of the wider community.

Micronutrient and inflammation indicator variables at baseline (early pregnancy, prior to supplement initiation) and late pregnancy (32 wk gestation, during supplementation) are presented by intervention group as mean ± SD and, for reference, as median (IQR) because data were often skewed. Micronutrient status indicators were first analyzed as continuous variables expressed on the arithmetic scale for interpretability, taking advantage of the large sample size. Log10-transformed data confirmed findings, as described later. Micronutrient status indicators were also used to categorize participants as deficient or not to calculate prevalence of deficiency.

Baseline comparability in indicators of micronutrient status and inflammation was ensured, initially using simple linear regression analysis with intervention as the independent variable (IFA = 0, MM = 1) and, second, with a common set of covariates added to that model for each indicator. Covariates included baseline characteristics that differed by intervention (LSI, age, height, and education categories) and conditions expected to influence micronutrient indicators regardless of underlying nutritional status [gestational age and season of blood draw (defined as hot–dry, February 16–June 15; monsoon, June 16–October 15; and winter, October 16–February 15) and, for micronutrients, AGP).

To test intervention efficacy on late pregnancy micronutrient status indicators, we used linear regression with intervention as the independent variable, adjusting for baseline indicator concentration (model 1). A second model (model 2) was generated using additional covariates, including the residuals of the corresponding baseline indicator data after regressing against gestational age, season of blood draw, and AGP values to remove their influences; LSI, age, height, and education categories; and late pregnancy AGP and gestational age and season at time of blood draw. The mean (95% CI) difference in indicator concentrations for MM compared with IFA from the β-coefficient for the intervention term is reported for both model 1 and model 2.

Intervention effects were confirmed by repeating model 2 using log10-transformed micronutrient status indicators. These models addressed skewness, lessening influence of extreme values, and allowed us to express differences in late pregnancy indicator values in the MM relative to the IFA group on a common scale (i.e., as percentage). Percentage difference was calculated as 100 × (10^β^ – 1), where β is the β-coefficient associated with the intervention term, taking advantage of the ability to express exponentiated differences in log10-transformed values as percentage. Minor differences in interpretation of the outcomes between the arithmetic and log10-transformed indicators are noted. Intervention differences are expressed graphically in relation to the percentage change in indicator values from baseline to late pregnancy, 1 of 2 post hoc analyses. Percentage change was calculated within intervention groups from the unadjusted difference of log10-transformed late pregnancy compared with baseline indicators, where mean percentage change (95% CI) was calculated as 100 × (10*^x^* – 1), with *x* being the mean (or lower or upper bound of the 95% CI) difference in log10 indicator values. The second post hoc analysis explored the relative contributions of *1*) the MM compared with IFA intervention; *2*) early pregnancy indicator concentrations; and *3*) other covariates combined in explaining late pregnancy indicator concentrations. This was done using the “hireg” command in STATA to build hierarchical regression models to test the contribution of each variable or set of variables to the total *R*^2^ and also the significance of the change in *R*^2^ with successive models.

Finally, a second preplanned analysis examined prevalence of abnormal indicators in late pregnancy by intervention group, after ensuring comparability of baseline status. The prevalence of micronutrient deficiencies in the IFA and MM groups is shown in early and late pregnancy, recognizing that indicator cutoffs may not be readily interpretable in late pregnancy. The prevalence rate ratio (PRR) and 95% CI of late pregnancy deficiency in the MM group relative to the IFA group were determined using the “glm” command in STATA, with a Poisson distribution, log-link, accounting for baseline status (as deficient = 1 or not = 0), and a similar set of covariates as described previously, to determine whether the MM intervention significantly reduced the prevalence of micronutrient deficiencies. The PRRs for being deficient in late pregnancy that were associated with having been deficient at baseline are also shown from the “glm” regression models.

Robust standard errors were generated for all regression models to account for cluster randomization. All data analysis was done using STATA version 13 (StataCorp); *P* < 0.05 was considered statistically significant, with some *P* < 0.10 noted.

## Results

**FIGURE 1:**
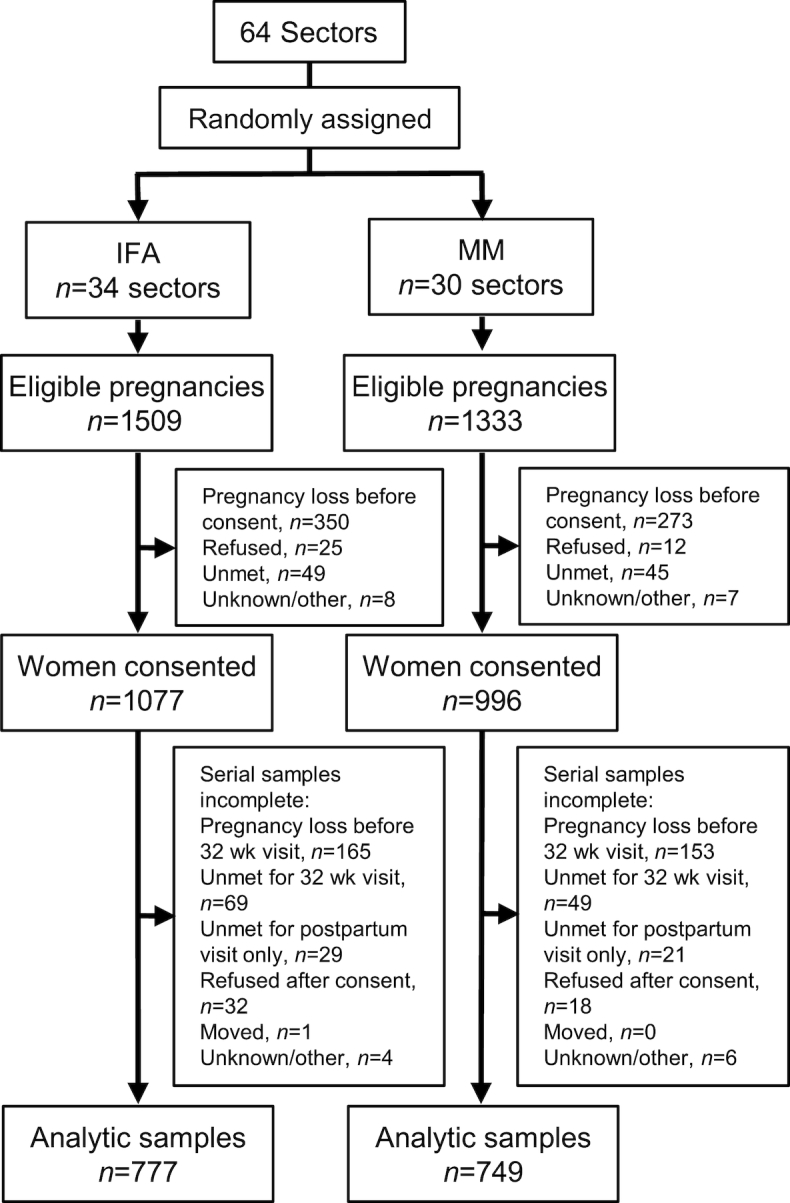
Consolidated Standards of Reporting Trials diagram for participants in the micronutrient status assessment substudy of the JiVitA-3 trial of antenatal multiple micronutrient (MM) compared with iron–folic acid (IFA) supplementation among women of rural Bangladesh. Pregnancy losses included those due to miscarriage, stillbirth, or elected pregnancy termination. Unmet refers to an inability to locate a woman in time to gain consent for or engage her participation in intended study visits.

Among 2842 detected pregnancies in 64 sectors, 2073 pregnant women were recruited, with few refusals(**Figure 1**). Pregnancy losses due to induced abortion, miscarriage, or stillbirth (2 cases only, because this is a late pregnancy event) prior to consent accounted for a substantial number of unenrolled cases (*n* = 350 for IFA, *n* = 273 for MM). Among consenting gravida, 547 (26%) lacked complete serial data, accounted for by pregnancy losses that occurred after the baseline visit, births that occurred before a late pregnancy visit could be scheduled, or losses to follow-up in the postpartum period. Serial samples from 1526 women remained for this analysis.

Substudy participants were younger, of lower parity, likely to be shorter, but better educated than participants in the main trial (*n* = 43,041) (2) (**Supplemental Table 1**). Fewer were enrolled before 8 wk gestation and during harvest season. There were no differences in LSI or diet and minor differences in morbidity between substudy and main trial participants. Adherence to intervention did not differ by substudy participation or supplement group (data not shown).

Baseline characteristics were similar between IFA and MM groups (**Table 1**). Nearly one-third of the women were <20 y old, and malnutrition by short stature (<150 cm) and underweight (BMI <18.5 kg/m^2^) was common. Despite differences in categorical distributions of age and height (Table 1), mean age (23.0 ± 5.4 compared with 22.9 ± 5.4 y, *P* = 0.81) and height (149.3 ± 5.4 compared with 148.9 ± 5.1 cm, *P* = 0.19) did not differ. Conversely, whereas percentage below median LSI did not differ (Table 1), mean LSI was somewhat higher for IFA compared with MM (*P* = 0.01). More than 60% of participants had ≥5 y of schooling, with ∼4% more reaching grade 10 in the IFA group. Nearly two-thirds of women reported eating ≥3 servings of fish in the last week, and ∼20% consumed ≥3 servings of yellow or green vegetables. Commonly reported morbidity symptoms included nausea, vomiting, low-grade fever, and productive cough. Gestational age at blood draw did not differ by intervention at baseline [median (IQR) for all: 10.0 (8.1, 12.9) wk] or late pregnancy [32.1 (31.9, 32.7) wk]. Season of baseline blood draw was comparable by intervention, whereas ∼4% fewer and ∼6% more IFA than MM participants had late pregnancy blood drawn during monsoon and winter seasons, respectively (*P* = 0.072 for chi-square analysis; not shown).

**TABLE 1:** Characteristics of pregnant women in rural Bangladesh at baseline assessment^1^

Characteristic		IFA (*n* = 777)	MM (*n* = 749)
Age at pregnancy,^2^ y	<20	235 (30.2)	254 (33.9)
	20–30	456 (58.7)	391 (52.2)
	≥30	86 (11.1)	104 (13.9)
Height,^2^ cm	<150	408 (52.5)	436 (58.3)
BMI, kg/m^2^	<18.5	308 (39.6)	324 (43.3)
Parity	0	303 (39)	295 (39.4)
	1	432 (55.6)	424 (56.6)
	≥2	42 (5.4)	30 (4.0)
Education completed,^2^ y	0	190 (24.5)	193 (25.8)
	1–4	98 (12.6)	103 (13.8)
	5–9	403 (51.9)	401 (53.5)
	≥10	86 (11.1)	52 (6.9)
LSI, median	Below	377 (48.5)	378 (50.5)
Diet, consumed ≥3 times in last wk	Meat	118 (15.2)	113 (15.1)
	Fish	513 (66.0)	486 (64.9)
	Eggs	146 (18.8)	130 (17.4)
	Milk	211 (27.2)	181 (24.2)
	Yellow vegetables	149 (19.2)	138 (18.4)
	Green vegetables	174 (22.4)	164 (21.9)
Morbidity, symptom present ≥1 d in last wk	Nausea	361 (46.5)	373 (49.8)
	Vomiting	182 (23.4)	204 (27.2)
	Low fever	261 (33.6)	228 (30.4)
	Cough	132 (17.0)	212 (16.2)
Gestational age at blood draw, wk	0 to <8	166 (21.5)	173 (23.2)
	8–12	399 (51.6)	404 (54.2)
	≥13	208 (26.9)	169 (22.7)
Season of blood draw^3^	Hot–dry	233 (30.0)	238 (31.8)
	Monsoon	339 (42.6)	304 (40.6)
	Winter	205 (26.4)	207 (27.6)

^1^Values are *n* (%). IFA, iron–folic acid; LSI, living standards index; MM, multiple micronutrient.

^2^Significantly different, *P* < 0.05 by chi-square test.

^3^Hot–dry, February 16–June 15; monsoon, June 16–October 15; winter, October 16–February 15.

Among the nutritional status indicators and AGP, no differences in mean ± SD (**Table 2**) concentrations between IFA and MM groups were observed at baseline, consistent with median (IQR) concentrations (**Supplemental Table 2**).

**TABLE 2:** Concentrations of micronutrient status indicators at baseline (presupplementation) and late (32 wk, postsupplementation) pregnancy and differences in mean indicator concentrations (MM compared with IFA) in late pregnancy among women of rural Bangladesh^1^

			IFA		MM		Concentration difference in late pregnancy: MM relative to IFA^2^					
							Model 1			Model 2		
Nutrient/condition	Indicator	Time	*n*	Mean ± SD	*n*	Mean ± SD	*n*	Mean (95% CI)	*P* value	*n*	Mean (95% CI)	*P* value
Anemia	Hb, g/L	BL	775	117.8 ± 10.6	747	117.7 ± 10.4	—	—		—	—	
		32 wk	775	111.2 ± 10.3	745	110.6 ± 10.3	1516	−0.6 (−1.6, 0.4)	0.219	1502	−0.5 (−1.4, 0.5)	0.341
Iron	Ferritin, µg/L	BL	757	74.8 ± 56.8	738	75.6 ± 58.4	—	—		—	—	
		32 wk	772	41.1 ± 36.2	740	37.8 ± 36.6	1481	−4.0 (−6.5, −1.4)	0.003	1471	−3.8 (−6.4, −1.3)	0.004
	TfR, mg/L	BL	256	4.9 ± 2.0	235	4.9 ± 2.2	—	—		—	—	
		32 wk	251	5.3 ± 2.3	233	5.4 ± 2.3	484	0.1 (−0.3, 0.5)	0.522	482	0.1 (−0.3, 0.5)	0.496
Folate	Plasma folate, nmol/L	BL	776	18.2 ± 9.1	747	17.5 ± 7.9	—	—		—	—	
		32 wk	774	30.6 ± 19.9	746	27.0 ± 20.3	1517	−3.2 (−6.7, 0.2)	0.064	1507	−2.6 (−5.8, 0.5)	0.103
Vitamin B-12	Cobalamin, pmol/L	BL	747	206 ± 104	727	200 ± 100	—	—		—	—	
		32 wk	686	164 ± 70	680	176 ± 70	1315	12 (6.1, 18.5)	<0.0001	1305	14 (8, 20)	<0.0001
Vitamin A	Retinol, µmol/L	BL	752	1.09 ± 0.29	725	1.07 ± 0.29	—	—		—	—	
		32 wk	774	0.99 ± 0.33	746	1.08 ± 0.33	1471	0.10 (0.07, 0.14)	<0.0001	1459	0.11 (0.07, 0.14)	<0.0001
Vitamin E	α-Toco, µmol/L	BL	754	12.0 ± 3.7	725	11.8 ± 3.7	—	—		—	—	
		32 wk	774	18.6 ± 5.2	746	19.4 ± 6.0	1473	1.0 (0.4, 1.7)	0.002	1461	1.0 (0.4, 1.5)	0.002
	α-Toco:Chol, µmol/mmol	BL	752	4.2 ± 1.2	719	4.1 ± 1.2	—	—		—	—	
		32 wk	774	4.3 ± 1.3	744	4.5 ± 1.8	1463	0.3 (0.1, 0.4)	0.005	1452	0.3 (0.1, 0.5)	0.002
	γ-Toco, µmol/L	BL	755	0.84 ± 0.59	726	0.84 ± 0.60	—	—		—	—	
		32 wk	774	1.06 ± 0.74	746	0.87 ± 0.66	1475	−0.18 (−0.27, −0.09)	<0.0001	1463	−0.14 (−0.22, −0.06)	0.001
Vitamin D	25(OH)D, nmol/L	BL	773	46.5 ± 13.7	748	47.2 ± 12.5	—	—		—	—	
		32 wk	774	46.3 ± 14.2	747	53.5 ± 16.2	1516	7.2 (5.2, 9.2)	<0.0001	1504	6.6 (4.8, 8.4)	<0.0001
Zinc	Plasma zinc, µmol/L	BL	762	11.3 ± 2.8	730	11.5 ± 2.8	—	—		—	—	
		32 wk	763	9.3 ± 2.4	741	9.7 ± 2.5	1471	0.4 (0.1, 0.6)	0.015	1461	0.3 (0.06, 0.6)	0.019
Iodine	Tg, µg/L	BL	729	9.2 ± 20.8	711	8.9 ± 17.2	—	—		—	—	
		32 wk	741	11.6 ± 22.6	726	9.7 ± 17.6	1407	−1.4 (−2.2, −0.7)	<0.0001	1397	−1.4 (−2.2, −0.7)	<0.0001
Inflammation	AGP, g/L	BL	775	0.77 ± 0.28	748	0.75 ± 0.29	—	—		—	—	
		32 wk	774	0.59 ± 0.24	747	0.57 ± 0.22	1518	−0.02 (−0.05, 0.01)	0.274	1510	−0.02 (−0.05, 0.01)	0.270

^1^AGP, α_1_-acid glycoprotein; BL, baseline; Hb, hemoglobin; IFA, iron–folic acid; LSI, living standards index; MM, multiple micronutrient; TfR, transferrin receptor; Tg, thyroglobulin; α-Toco, α-tocopherol; α-Toco:Chol, α-tocopherol:cholesterol ratio; γ-Toco, γ-tocopherol; 25(OH)D, 25-hydroxyvitamin D.

^2^Baseline indicators did not differ by intervention status in either unadjusted models or after adjustment for LSI, age and education categories, gestational age and season of blood draw and, for micronutrient indicators, AGP (not shown). Late pregnancy differences in indicators in MM relative to IFA are shown in model 1, based on linear regression models of late pregnancy indicator values adjusting only for baseline indicator concentrations, or model 2, adjusting for baseline indicator data as residuals after regressing against gestational age, season, and AGP values to remove their influences; LSI, age, height, and education categories; gestational age and season of follow-up blood draw; and late pregnancy AGP for micronutrient status indicators. *F*-statistics were *P* < 0.0001 for all models except model 1 for TfR (*P* = 0.0282) and zinc (*P* = 0.0235).

In late pregnancy (Table 2), despite receiving the same dosage of iron and folic acid, mean ferritin was lower among women in the MM group than those in the IFA group, with a similar tendency for folate (not significant). For other micronutrients, MM supplementation increased plasma cobalamin (B-12), retinol, α-tocopherol and α-tocopherol:cholesterol, 25(OH)D, and zinc relative to IFA. Mean γ-tocopherol and Tg were lower in MM than in IFA supplemented women, the latter consistent with better iodine status in the MM group. There was no difference in mean AGP by intervention. Findings were similar between model 1 and model 2, demonstrating robustness of the MM impact.

**FIGURE 2:**
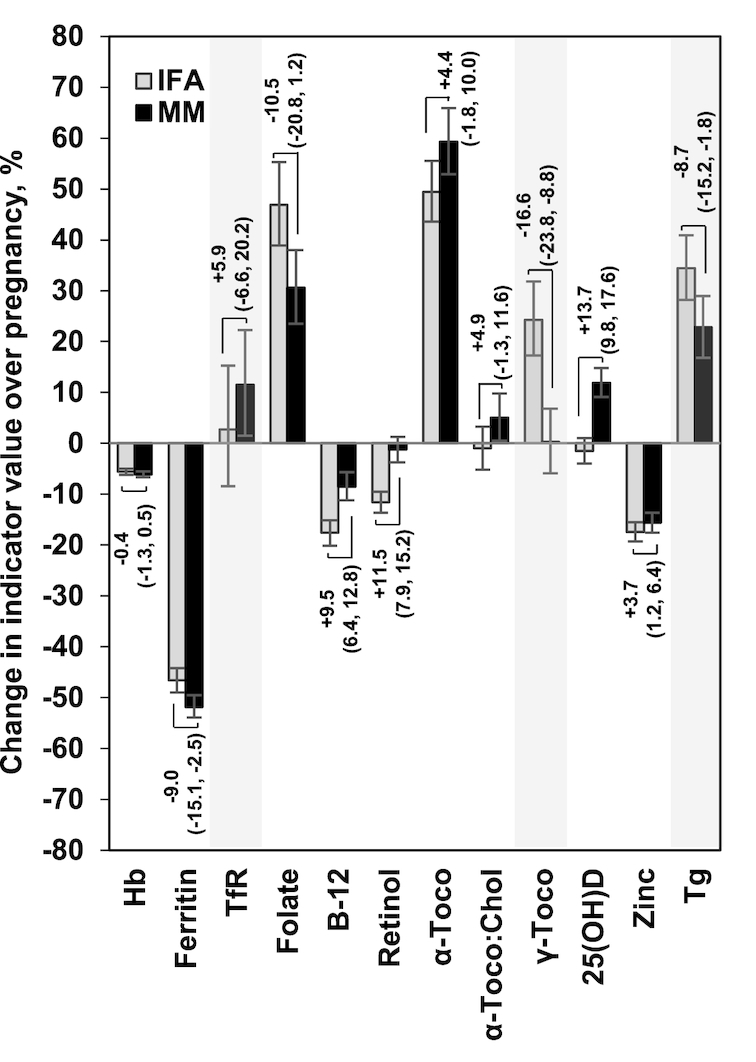
Percentage (95% CI) difference in micronutrient status indicators at 32 wk gestation in MM compared with IFA recipients (represented by brackets) in the context of pregnancy-associated changes in micronutrient status indicators expressed as percentage change (95% CI) from baseline (bars). Differences in MM versus IFA are derived from adjusted (model 2 from Table 2 using log10-transformed indicators), and percentage change over pregnancy from unadjusted, analyses. For example, there was no significant difference in Hb in late pregnancy between intervention groups (0.4% lower in MM than in IFA), while Hb was ∼6% lower in late compared to early pregnancy in both IFA and MM recipients. Ferritin was 9.0% lower among the MM than IFA recipients, and declined by 47% with IFA and 52% with MM from baseline to 32 wk gestation. Shaded indicators are those for which higher concentrations represent poorer status. Hb, hemoglobin; IFA, iron–folic acid; MM, multiple micronutrients; TfR, transferrin receptor; Tg, thyroglobulin; α-Toco, α-tocopherol; α-Toco:Chol, α-tocopherol:cholesterol ratio; γ-Toco, γ-tocopherol; 25(OH)D, 25-hydroxyvitamin D.

Expressed as a percentage difference in plasma indicators in late pregnancy (**Figure 2**), the MM relative to IFA supplement had no impact on Hb (*P* = 0.365) but decreased ferritin by 9.0% (*P* = 0.008) in the MM group relative to IFA group, with little impact on TfR (*P* = 0.364) and a nonsignificant but notable decrease in folate (*P* = 0.077) with MM. Increases in vitamin B-12, retinol, and 25(OH)D (all *P* < 0.0001), of a similar magnitude to the declines in ferritin and folate, were observed with MM compared with IFA. Relative increases in α-tocopherol (*P* = 0.164) and α-tocopherol:cholesterol (*P* = 0.124) with MM were not significant when assessed with log10-transformed indicators, but the decline in γ-tocopherol with MM compared with IFA (*P* < 0.0001) remained substantial. More modest differences in plasma zinc (*P* = 0.005) and Tg (*P* = 0.016) still favored improved status with MM.

Figure 2 shows the intervention effects just mentioned in relation to pregnancy-associated percentage changes in indicators observed in the post hoc, unadjusted analysis. Regardless of intervention, indicator values declined significantly over pregnancy for Hb, ferritin, vitamin B-12, and zinc; conversely, plasma folate, α-tocopherol, and Tg (toward deficiency) increased. Increases occurred only with MM for TfR (toward deficiency), α-tocopherol:cholesterol, and 25(OH)D, and MM prevented a decline in retinol. An increase in γ-tocopherol from early pregnancy occurred only with IFA.

**FIGURE 3:**
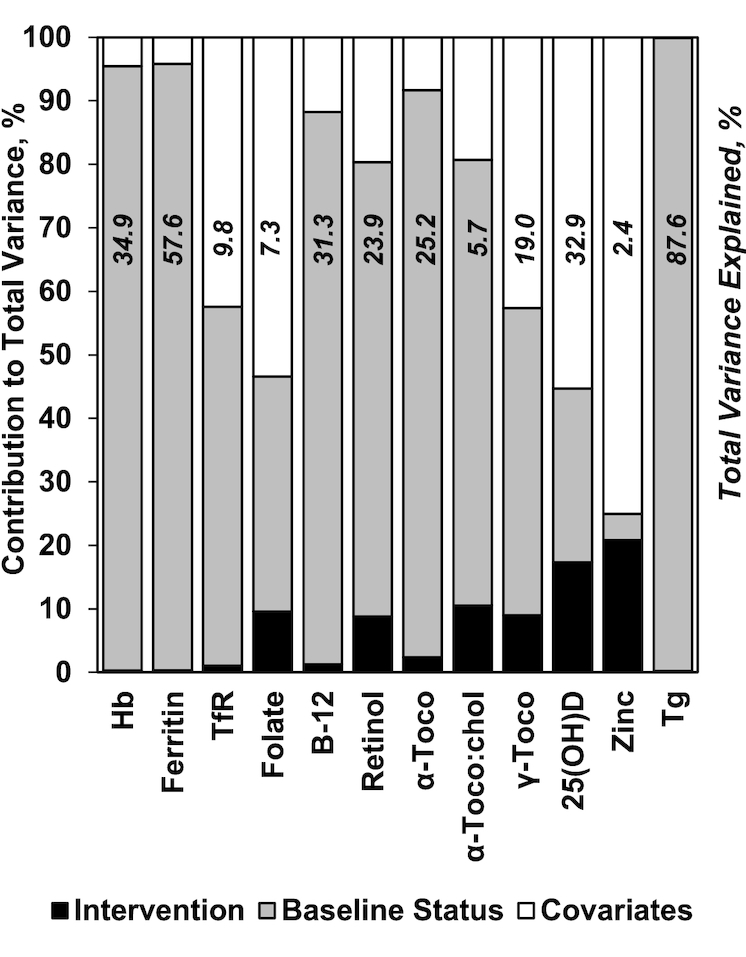
Percentage contribution of intervention (MM compared with IFA), baseline micronutrient status indicator concentrations (accounting for season, gestational age, and AGP concentration), and other covariates (including factors that differed at baseline and season, gestational age, and AGP concentration at the time of the 32-wk measurement) to the total variance in late pregnancy micronutrient indicator concentrations among women in rural Bangladesh. Data are from regression model 2 in Table 2. Also shown is the total explained variance (*R*^2^), in italics, of 32-wk micronutrient indicator concentrations based on model 2. AGP, α_1_-acid glycoprotein; Hb, hemoglobin; IFA, iron–folic acid; MM, multiple micronutrient; TfR, transferrin receptor; Tg, thyroglobulin; α-Toco, α-tocopherol; α-Toco:Chol, α-tocopherol:cholesterol ratio; γ-Toco, γ-tocopherol; 25(OH)D, 25-hydroxyvitamin D.

Contributions of the MM intervention, baseline indicator concentrations, and other covariates to each late pregnancy indicator were explored in the second post hoc analysis (**Figure 3**). Baseline indicator concentrations typically explained most of the variance in late pregnancy indicators. Exceptions included folate, and 25(OH)D, for which variance was largely explained by season of analysis, and zinc, for which intervention and other covariates prevailed. The total explained variance in late pregnancy indicators was least for zinc (2.4%), α-tocopherol:cholesterol, folate, and TfR, but was 87.6% for thyroglobulin, reflecting nearly complete concurrence with prior status. Explained variance ranged from 19.0% (α-tocopherol) to 57.6% (ferritin) for other indicators.

There were no differences by intervention in baseline prevalence of deficiency (**Table 3**), which among all women was for anemia, 20.6%; iron by ferritin, 4.0%; iron by TfR, 4.7%; folate, 2.5%; vitamin B-12, 35.4%; vitamin A, 6.7% and 48.3% by retinol <0.70 and <1.05 µmol/L, respectively, and 41.0% with low β-carotene; vitamin E, 57.7%; vitamin D, 64.0%; zinc, 13.4%; and iodine, 2.6%. Almost all women (93.6%) entered pregnancy with at least 1 deficiency, and 67.2% had ≥2 abnormal indicators (not shown). Baseline AGP was elevated in 17.8% and 15.1% of women by IFA and MM, respectively (*P* = 0.072).

**TABLE 3:** Baseline (presupplementation) and late (32 wk, postsupplementation) pregnancy prevalence of deficiency and the prevalence rate ratio for micronutrient deficiency in late pregnancy by intervention status among women of rural Bangladesh^1^

		IFA		MM		PRR for late pregnancy deficiency^2^				
							MM vs. IFA		Baseline deficiency vs. sufficiency	
Indicator	Time	*n*	% Affected	*n*	% Affected	*n*	PRR (95% CI)	*P* value	PRR (95% CI)	*P* value
Hb <110 g/L	BL	775	21.0	747	20.1	—	—		—	
	32 wk	775	42.6	745	42.8	1505	1.01 (0.89, 1.14)	0.899	2.04 (1.82, 2.29)	<0.0001
Ferritin <15 µg/L	BL	757	4.1	738	3.8	—	—		—	
	32 wk	772	17.2	740	19.7	1472	1.14 (0.85, 1.51)	0.382	3.70 (2.84, 4.81)	<0.0001
TfR >8.3 mg/L	BL	256	6.3	235	3.0	—	—		—	
	32 wk	251	10.0	233	11.2	482	1.15 (0.70, 1.91)	0.579	2.06 (0.86, 4.98)	0.107
Plasma folate <6.8 nmol/L	BL	776	2.3	747	2.7	—	—		—	
	32 wk	774	5.8	746	7.0	1508	1.14 (0.75, 1.74)	0.545	4.82 (2.82, 8.24)	<0.0001
Vitamin B-12 <150 pmol/L	BL	747	34.1	727	36.6	—	—		—	
	32 wk	686	52.0	680	42.9	1306	0.81 (0.71, 0.93)	0.002	2.13 (1.94, 2.35)	<0.0001
Retinol <0.70 µmol/L	BL	752	6.3	725	7.2	—	—		—	
	32 wk	774	18.7	746	11.9	1461	0.62 (0.50, 0.78)	<0.0001	3.68 (2.91, 4.67)	<0.0001
Retinol <1.05 µmol/L	BL	752	46.5	725	50.2	—	—		—	
	32 wk	774	60.9	746	48.7	1461	0.78 (0.71, 0.85)	<0.0001	1.65 (1.50, 1.81)	<0.0001
α-Toco <12 µmol/L	BL	754	56.6	725	58.8	—	—		—	
	32 wk	774	6.6	746	7.4	1463	1.13 (0.77, 1.66)	0.542	3.67 (2.27, 5.94)	<0.0001
α-Toco:Chol <2.2 µmol/mmol	BL	752	2.5	719	3.2	—	—		—	
	32 wk	774	2.7	745	3.2	1456	1.24 (0.69, 2.26)	0.472	3.08 (1.05, 9.05)	0.040
25(OH)D <50 nmol/L	BL	773	65.2	748	62.7	—	—		—	
	32 wk	774	64.1	747	45.8	1507	0.74 (0.66, 0.83)	<0.0001	1.42 (1.28, 1.57)	<0.0001
Plasma zinc <9.0 µmol/L	BL	762	14.4	730	12.3	—	—		—	
	32 wk	763	51.8	741	43.3	1462	0.85 (0.75, 0.95)	0.006	1.17 (1.01, 1.36)	0.031
Tg >40 µg/L	BL	729	2.6	711	2.5	—	—		—	
	32 wk	741	3.8	726	2.2	1398	0.66 (0.41, 1.07)	0.094	62.8 (32.6, 121.2)	<0.0001
AGP >1.0 g/L	BL	775	17.8	748	15.1	—	—		—	
	32 wk	774	6.7	747	4.0	1510	0.58 (0.35, 0.96)	0.033	1.90 (1.16, 3.09)	0.010

^1^AGP, α_1_-acid glycoprotein; BL, baseline; Hb, hemoglobin; IFA, iron–folic acid; LSI, living standards index; MM, multiple micronutrient; PRR, prevalence rate ratio; TfR, transferrin receptor; Tg, thyroglobulin; α-Toco, α-tocopherol; α-Toco:Chol, α-tocopherol:cholesterol ratio; 25(OH)D, 25-hydroxyvitamin D.

^2^No significant differences in early pregnancy prevalence of deficiency occurred between MM and IFA based on unadjusted or adjusted (for LSI; age, height, and education categories; gestational age; season of blood draw; and inflammation) “glm” regression models using Poisson distributions, a log-link function, and robust standard errors to account for cluster randomization. Intervention effects in late pregnancy were based on similar models adjusted for baseline status (deficient or not; shown in the table); LSI; age, height, and education categories; gestational age; season; and the presence of inflammation at the time of late pregnancy blood draw. Robust standard errors were calculated to account for cluster randomization. Combined effects of MM and early pregnancy status on the prevalence of deficiency relative to IFA and early pregnancy sufficiency (as the referent conditions) can be estimated as the product of the PRR associated with MM supplement receipt and baseline deficiency.

In the primary analysis of the intervention efficacy in affecting prevalence of deficiency, PRRs were consistent with lower prevalence of deficiency in the MM relative to the IFA supplementation group for vitamin B-12 (e.g., 19% lower), vitamin A as retinol <0.70 or <1.05 µmol/L, vitamin D, zinc, and inflammation (Table 3).

In both intervention groups, there were apparent increases in deficiency from baseline to 32 wk pregnancy for most micronutrients. Exceptions were 25(OH)D (prevalence declined in MM only), Tg (low prevalence at baseline and little apparent change), and α-tocopherol (apparent deficiency markedly decreased in both groups). The PRRs for being deficient in later pregnancy typically were 2–5 times higher among women who entered pregnancy in a deficient state; exceptions were retinol <1.05 µmol/L, 25(OH)D, and zinc. The large point estimate for Tg was explained by late pregnancy deficiency nearly universally occurring among those deficient at baseline.

Combined effects of MM and early pregnancy deficiency relative to IFA and early pregnancy sufficiency (as the referent conditions) can be estimated as the product of the PRR associated with MM supplement receipt and baseline deficiency in Table 3. For example, although the MM reduced vitamin B-12 deficiency by 19% overall, MM recipients who started pregnancy in a deficient state still had 1.72 times the risk of being considered deficient in late pregnancy compared with women receiving IFA who began their pregnancies in a sufficient state (1.72 = 0.81 × 2.13 for MM compared with IFA and deficiency compared with sufficiency, respectively).

## Discussion

Multiple micronutrient supplementation relative to IFA alone improved status of micronutrients other than iron and folate in pregnant women in rural Bangladesh in a randomized, controlled trial in which MM extended gestational age and increased infant birth size relative to IFA (2). The trial took place among community-dwelling women in an area typical of rural Bangladesh (32) and, more broadly, rural Gangetic South Asia. Women nearly universally entered pregnancy with at least 1 deficiency, consistent with the overall poor diet, short stature, low BMI, and food insecurity observed in this population (33). However, deficiencies persisted despite benefits of antenatal MM supplementation.

Women began pregnancies replete in iron, despite prevalent anemia, consistent with national survey data (34). In Bangladesh, iron sufficiency has been associated with high groundwater and tube well water iron content (35–38) and low Hb with hemoglobinopathies (35) and lower plasma zinc, vitamin B-12, and α-tocopherol concentrations (39). Despite provision of iron to all women, anemia and iron deficiency by TfR nearly doubled, and low iron stores quadrupled over pregnancy, consistent with high pregnancy iron requirements and prioritization for the fetus. A similar magnitude of decline in ferritin over pregnancy was observed by Ziaei et al. (14) in a trial in Bangladesh comparing MM to IFA regimens with either 30 or 60 mg of iron, with no differences in ferritin observed by intervention group. Greater declines in ferritin in the MM group in the current study suggest competition for absorption (e.g., with zinc) or enhanced utilization of iron with MM compared with IFA.

Folate deficiency was uncommon at pregnancy outset despite low reported intake of green leafy vegetables, perhaps from consumption of pulses and other compensatory foods. Circulating folate increased over pregnancy with both IFA and MM, similar to findings of Ziaei et al. (14), although deficiency prevalence also doubled. We could not discern forms of circulating folate but assume 5-methyltetrahydrafolate would predominate (40, 41). More efficient tissue uptake and utilization of circulating folate, explaining the ∼10% lower (albeit not significant) folate in MM recipients, may have occurred with provision of vitamin B-12 in the MM supplement (42).

Little folate deficiency with extensive vitamin B-12 deficiency has been linked to poor fetal growth and development and enhanced risk of chronic disease in South Asia (25, 43), potentially through alterations in 1-carbon metabolism (42). Despite benefits of MM on vitamin B-12 status, circulating cobalamin declined over pregnancy in both IFA and MM groups, as described by others (14, 15, 44). Among Indian women with an ∼50% prevalence of deficiency, antenatal 50 µg vitamin B-12 supplements, nearly 20 times the dose in the MM supplement, prevented a decline in vitamin B-12 from early to late pregnancy (45). Those findings suggest that considerably more than 1 RDA would be required to eliminate apparent vitamin B-12 deficiency during pregnancy in this setting, although low circulating B-12 during pregnancy may reflect a redistribution of the nutrient, for example, to the fetus (14, 15, 44).

Vitamin A deficiency was <10% at the baseline assessment, consistent with recent national survey data (34). However, >40% of women had marginal status and circulating β-carotene was low, suggesting risk for deficiency (46). The MM supplement prevented a decline in retinol over pregnancy, as previously observed (16, 17). There was a greater reduction in prevalence of deficiency among those who started their pregnancies in a deficient rather than a marginal-to-deficient state, yet baseline deficiency remained a strong predictor of late pregnancy deficiency.

The majority (∼60%) of women entered pregnancy deficient in vitamins D and E. Vitamin D deficiency has been observed in women of reproductive age in Bangladesh (47), and it confers risk of deficiency to infants (48, 49). Circulating 25(OH)D did not change over pregnancy among women receiving IFA, but MM resulted in higher 25(OH)D and a 26% reduction in prevalence of deficiency relative to IFA, despite a low daily dose of vitamin D (5 µg/d) compared to current recommendations (15 µg/d) (30). Despite MM benefits, nearly half of recipients remained deficient. A small placebo-controlled trial among pregnant Bangladeshi women showed that a weekly dose of 35,000 IU (875 µg) of vitamin D in late pregnancy improved maternal and infant status and eliminated vitamin D deficiency without demonstrable risk of harm (50). A systematic review reported benefits of vitamin D supplementation on status and pregnancy outcomes, but heterogeneity in supplement amount and regimen among trials is extensive (51), reinforcing a need for consensus on vitamin D recommendations in pregnancy.

α-Tocopherol is the preferred analyte for assessing vitamin E deficiency in undernourished populations (52), but it increases in proportion to circulating lipids as pregnancy advances (20). We attempted to account for this using α-tocopherol:cholesterol, but the near absence of deficiency with the ratio suggests a lack of validity for its use to detect deficiency in this context. There was a modest response of both α-tocopherol indicators to the MM supplement, although it was nonsignificant when assessed in log10-transformed data, suggesting the MM effect occurred in the upper tail of the α-tocopherol distribution. More notable was the prevention of increased γ-tocopherol with MM, consistent with findings from other studies (10, 53, 54). Elevated γ-tocopherol is associated with poorer health practices (53–55), miscarriage (56), and preterm birth (57), so the MM impact on γ-tocopherol may be particularly important.

Among minerals, zinc deficiency in early pregnancy was lower than expected (∼13%) based on experience (10) and regional evidence (34, 58). The MM supplement led to a small but significant increase in circulating zinc, reducing deficiency by 16%, with little impact of baseline status on late pregnancy status. Our findings contrast with a lack of impact on zinc of an antenatal MM intervention in rural Nepal, where deficiency was more common (10), and elsewhere in Bangladesh (14). Iodine deficiency by elevated Tg was low (∼2.5%) and remained so throughout pregnancy, but it persisted among women who entered pregnancy deficient. Low prevalence of iodine deficiency is inconsistent with data showing moderate iodine deficiency in Bangladesh using urinary iodine (59). Thyroglobulin might better indicate individual status than urinary iodine, intended for population assessment (31, 59). However, use of Tg has not been reported often during pregnancy, with this being one of the largest studies to assess Tg response to iodine supplementation (19). Higher Tg in late pregnancy, somewhat ameliorated in the MM group, is consistent with enhanced demand for iodine.

Prevalence of inflammation (∼15%) in early pregnancy was lower than the occurrence of most commonly reported morbidity symptoms, and AGP concentrations declined during pregnancy. The MM supplement reduced inflammation in late pregnancy, although its prevalence was low. Inclusion of AGP in adjusted regression models distinguished a role for the MM supplement in improving micronutrient status through mechanisms other than reduced inflammation.

Despite MM benefits, baseline status was typically the strongest determinant of late pregnancy deficiencies, suggesting benefits of entering pregnancy in a replete state. Vitamins and minerals in amounts of ∼1 RDA were typically insufficient to overcome pre-existing deficiencies. Determining postpartum status (data not yet available) would show accrued benefits of supplementation and overcome challenges in interpreting indicators in pregnancy, when nutrient demands, plasma volume expansion, and metabolic adaptations may influence them. However, pregnancy status may be particularly relevant for maternal, birth, and infant outcomes. Yet, variability in pregnancy-associated changes in indicators demonstrates that a common approach for addressing status assessment in pregnancy is not feasible. Rather, each micronutrient needs to be understood for its own metabolic pathways, impact of conditions such as gestational age and inflammation, and even unique features of a population—given, for example, the limited plasma volume expansion among these Bangladeshi women (12) compared with others (13). Particularly low explained variance of some indicators also suggests that more research is warranted on indicator determinants, validity for describing status, and responsiveness to interventions.

Limitations of this study include the inability to assess the status of all micronutrients in the MM supplement or to utilize the most comprehensive indicators for some (e.g., plasma folate types or RBC folate, or methylmalonic acid for vitamin B-12), although this would have been cost prohibitive. Strengths include the randomized design, high adherence, large sample size, community-based assessments, samples collected at narrowly defined time points including immediately after pregnancy ascertainment, and the number of micronutrients assessed.

Poor status was most common for micronutrients other than iron and folate, providing a strong impetus for provision of nutritional support beyond IFA. Earlier intervention or higher micronutrient contents are warranted to prepare women for the nutritional demands of pregnancy in settings in which micronutrient deficiencies are common, wide-ranging, and confer risk of adverse pregnancy outcomes to women and their offspring.

## Supplementary Material

nxz046_Supplemental_Files

## Notes

The JiVitA-3 trial was funded through grant GH614 (Global Control of Micronutrient Deficiency) from the Bill and Melinda Gates Foundation (Ellen Piwoz, Senior Program Officer). Additional assistance was received from the Sight and Life Research Institute. DSM N.V. provided in-kind support by formulating and delivering micronutrient premixes for supplement production and testing for potency gratis. Beximco Pharmaceuticals produced, bottled, labeled, and delivered supplement tablets to the field site gratis.

Author disclosures: KJS, SM, SS, HA, AAS, LS-FW, MM, MAA, BK, PS, EU, RM, RDWK, BU, ABL, KPW, and PC, no conflicts of interest.

Supplemental Tables 1 and 2 are available from the “Supplementary data” link in the online posting of the article and from the same link in the online table of contents at https://academic.oup.com/jn/.

Abbreviations used: AGP, α_1_-acid glycoprotein; Hb, hemoglobin; IFA, iron–folic acid; LSI, living standards index; MM, multiple micronutrient; PRR, prevalence rate ratio; TfR, transferrin receptor; Tg, thyroglobulin; 25(OH)D, 25-hydroxyvitamin D.
