# Supplementary material for: Antenatal Multiple Micronutrient Supplementation Compared to Iron–Folic Acid Affects Micronutrient Status but Does Not Eliminate Deficiencies in a Randomized Controlled Trial Among Pregnant Women of Rural Bangladesh
Source: J Nutr. 2019 Apr 22;149(7):1260–70. doi: 10.1093/jn/nxz046 (PMC6602890; doi:10.1093/jn/nxz046)
Supplement: nxz046_Supplemental_Files [file nxz046_supplemental_files.zip › Supplementary Data_Table 1.pdf]

Supplementary Data

Supplemental Table 1. Comparison of enrollment characteristics of pregnant women in the micronutrient status assessment substudy compared to all other participants in the JiVitA-3 antenatal IFA and MM intervention trial in rural Bangladesh[1]

Indicator		Substudy (*n*=1526)	Main Trial (*n*=43041)	p-value
Age at pregnancy[2], y	<20	489 (32.0)	11788 (27.4)	
	20-30	847 (55.5)	21980 (51.1)	
	≥30	190 (12.5)	9255 (21.5)	<0.0001
Height[2], cm	<150	844 (55.3)	18964 (52.2)	0.0168
BMI, kg/m^2	<18.5	632 (41.4)	14554 (40.1)	0.2925
Parity	0	598 (39.2)	12720 (29.6)	
	1	856 (56.1)	25919 (60.4)	
	≥2	72 (4.7)	4308 (10.0)	<0.0001
Education completed[2], y	0	383 (25.1)	13157 (30.6)	
	1-4	201 (13.2)	6311 (14.7)	
	5-9	804 (52.7)	20345 (47.4)	
	≥10	138 (9.0)	3123 (7.3)	<0.0001
LSI, median	Below	755 (49.5)	21558 (50.2)	0.5670
Diet, Consumed of ≥ 3 times in last wk	Meat	231 (15.1)	5350 (14.7)	0.6356
	Fish	999 (65.5)	23329 (64.1)	0.2745
	Eggs	276 (18.1)	6378 (17.5)	0.5717
	Milk	392 (25.7)	9367 (25.7)	0.9663
	Yellow vegetable	287 (18.8)	6960 (19.1)	0.7579
	Green vegetable	338 (22.2)	8093 (22.2)	0.9352
Morbidity, Symptom present ≥1 day in last wk	Nausea	734 (48.1)	17024 (46.8)	0.3109
	Vomiting	386 (25.3)	8356 (23.0)	0.0340
	Low fever	489 (32.0)	12100 (33.3)	0.3259
	Cough	253 (16.6)	5091 (14.0)	0.0044
Gestational age at enrollment (Baseline), wk	0-<8	361 (23.8)	12237 (28.8)	
	8-12	787 (51.9)	21297 (50.0)	
	≥13	369 (24.3)	9029 (21.2)	<0.0001
Season of Enrollment[3] (Baseline)	Hot-dry	468 (30.7)	18360 (42.7)	
	Monsoon	639 (41.9)	13530 (31.4)	
	Winter	419 (27.4)	11148 (25.9)	<0.0001

[1] Values are *n* (%); LSI, living standards index.

[2] Significantly different, $P < 0.05$ by Chi-squared test.

[3] Hot-dry, February 16-June 15; Monsoon, June 16-October 15; Winter, October 16-February 15.
