# Supplementary material for: Antenatal Multiple Micronutrient Supplementation Compared to Iron–Folic Acid Affects Micronutrient Status but Does Not Eliminate Deficiencies in a Randomized Controlled Trial Among Pregnant Women of Rural Bangladesh
Source: J Nutr. 2019 Apr 22;149(7):1260–70. doi: 10.1093/jn/nxz046 (PMC6602890; doi:10.1093/jn/nxz046)
Supplement: nxz046_Supplemental_Files [file nxz046_supplemental_files.zip › Supplementary Data_Table 2.pdf]

Supplementary Data

Supplemental Table 2. Concentrations of micronutrient status indicators at baseline and 32 wk by antenatal supplement intervention groups among women of rural Bangladesh[1]

Nutrient	Indicator	Time	IFA		MM	
			n	Median (IQR)	*n*	Median (IQR)
Iron	Hb, g/L	BL	775	118 (111, 125)	747	118 (111, 125)
		32 wk	773	111 (105, 118)	743	111 (105, 117)
	Ferritin, µg/L	BL	757	62.8 (38.7, 93.5)	738	62.5 (39.3, 93.4)
		32 wk	752	32.0 (20.8, 48.9)	729	29.8 (19.2, 43.5)
	TfR, mg/L	BL	256	4.6 (3.6, 5.8)	235	4.4 (3.6, 5.8)
		32 wk	251	4.9 (3.9, 6.3)	233	4.9 (3.9, 6.4)
Folate	Total plasma folate, nmol/L	BL	776	16.5 (12.0, 22.1)	747	16.5 (12.3, 21.1)
		32 wk	773	27.3 (13.8, 44.2)	744	20.8 (12.1, 38.1)
Vitamin B-12	Total cobalamin, pmol/L	BL	747	181 (132, 243)	727	171 (134, 239)
		32 wk	656	148 (118, 191)	659	159 (130, 204)
Vitamin A	Retinol[4], µmol/L	BL	752	1.08 (0.89, 1.27)	725	1.05 (0.87, 1.24)
		32 wk	749	0.96 (0.76, 1.19)	722	1.06 (0.84, 1.28)
Vitamin E	α-Toco, µmol/L	BL	754	11.6 (9.5, 13.9)	725	11.3 (9.5, 13.5)
		32 wk	751	18.4 (15.7, 21.6)	722	19.2 (16.1, 23.0)
	α-Toco:Chol, µmol/mmol	BL	752	4.02 (3.40, 4.77)	719	3.97 (3.34, 4.62)
		32 wk	748	4.22 (3.50, 5.01)	714	4.29 (3.64, 5.24)
	γ-Toco, µmol/L	BL	755	0.70 (0.44, 1.04)	726	0.67 (0.44, 1.06)
		32 wk	752	0.86 (0.54, 1.35)	723	0.68 (0.420, 1.12)
Vitamin D	25(OH)D, nmol/L	BL	773	44.8 (38.1, 53.8)	748	45.6 (38.8, 53.9)
		32 wk	770	44.9 (35.7, 53.8)	746	51.7 (42.0, 63.2)
Zinc	Total plasma zinc, µmol/L	BL	762	10.7 (9.5, 12.5)	730	11.0 (9.8, 12.5)
		32 wk	748	8.9 (7.9, 10.2)	723	9.3 (8.2, 10.7)
Iodine	Tg, µg/L	BL	729	5.5 (2.6, 9.5)	711	5.4 (2.8, 9.8)
		32 wk	710	7.3 (3.7, 11.5)	697	6.4 (3.6, 10.7)
Inflammation	AGP, g/L	BL	775	0.74 (0.57, 0.92)	748	0.71 (0.56, 0.88)
		32 wk	774	0.55 (0.45, 0.70)	747	0.54 (0.43, 0.66)

[1]AGP, α-1 acid glycoprotein; BL, baseline; Hb, hemoglobin; IFA, iron folic acid; MM, multiple micronutrient; TfR, transferrin receptor; Tg, thyroglobulin; α-Toco, α-tocopherol; α-Toco:Chol, α-tocopherol:cholesterol ratio; γ-Toco, γ-tocopherol; 25(OH)D, 25-hydroxyvitamin D.
